# Supplementary material for: Aromatic and arginine content drives multiphasic condensation of protein-RNA mixtures
Source: Biophys J. 2023 Jul 5;123(11):1342–55. doi: 10.1016/j.bpj.2023.06.024 (PMC11163273; doi:10.1016/j.bpj.2023.06.024)
Supplement: Document S1. Figures S1–S8 [file mmc1.pdf]

Biophysical Journal, Volume *123*

Supplemental information

Aromatic and arginine content drives multiphasic condensation of protein-RNA mixtures

Pin Yu Chew, Jerelle A. Joseph, Rosana Collepardo-Guevara, and Aleks Reinhardt

Supporting Material:
Aromatic and arginine content drives multiphasic condensation of protein–RNA mixtures

Pin Yu Chew,[1] Jerelle A. Joseph,[2] Rosana Collepardo-Guevara,[1,3,4,a)] and Aleks Reinhardt[1,b)]

[1)] *Yusuf Hamied Department of Chemistry, University of Cambridge, Cambridge, CB2 1EW, United Kingdom*

[2)] *Department of Chemical and Biological Engineering, Princeton University, Princeton, NJ 08544, USA*

[3)] *Department of Physics, University of Cambridge, Cambridge, CB3 0HE, United Kingdom*

[4)] *Department of Genetics, University of Cambridge, Cambridge, CB2 3EH, United Kingdom*

(Dated: 20 June 2023)

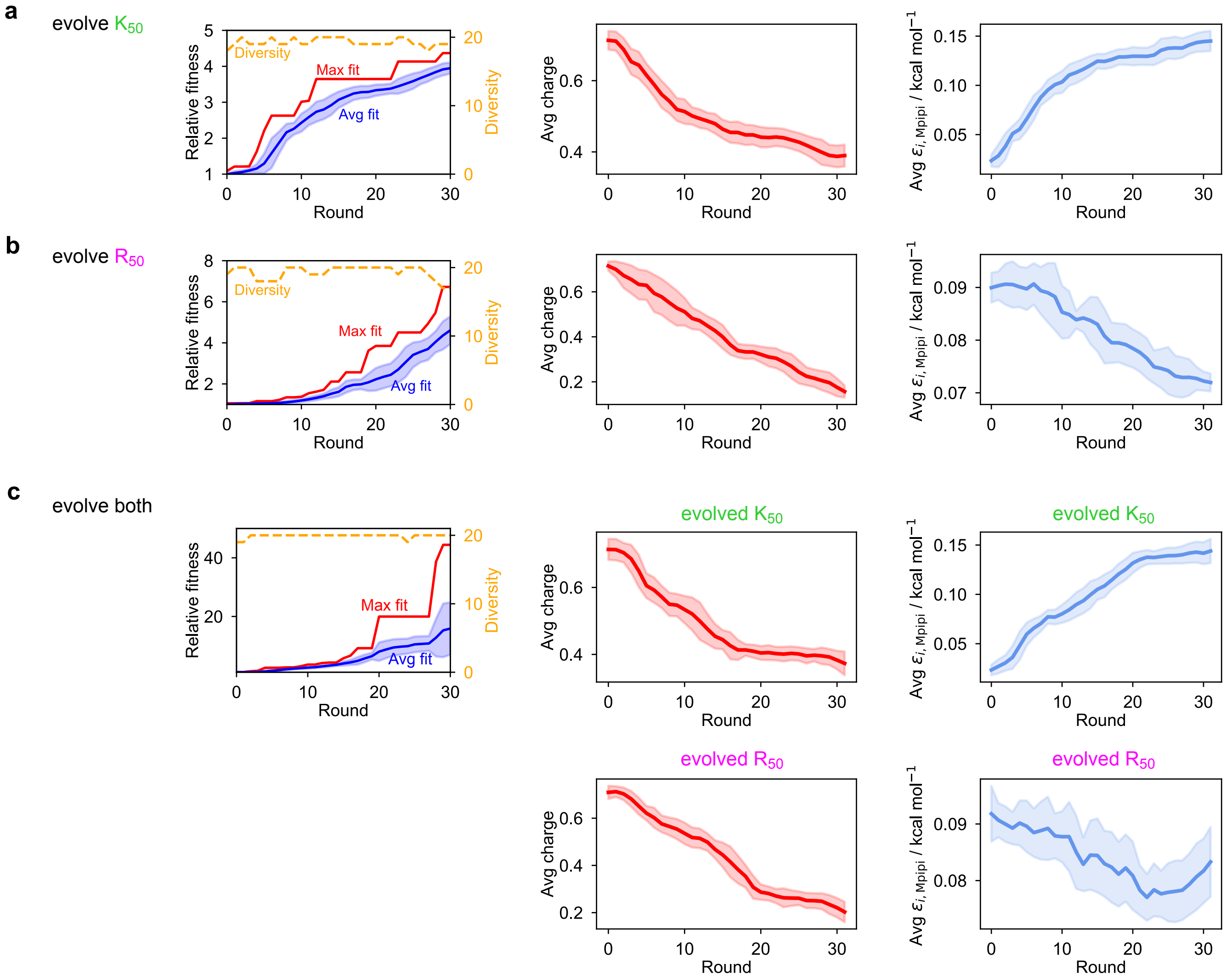

Figure S1. Genetic-algorithm progressions and changes in average charge and $\varepsilon_{i,\mathrm{Mpipi}}$ over the residues of the evolved sequence(s) as a function of the round number for the genetic-algorithm runs towards decreasing multiphasicity, where we evolve (*a*) K_{50} or (*b*) R_{50} in separate runs, or (*c*) both sequences simultaneously. For (*c*), the change in average charge and $\varepsilon_{i,\mathrm{Mpipi}}$ are given separately for both protein sequences. Shaded areas correspond to the standard deviation across all 20 sequences in the population at each round.

[a)] Electronic mail: rc597@cam.ac.uk

[b)] Electronic mail: ar732@cam.ac.uk

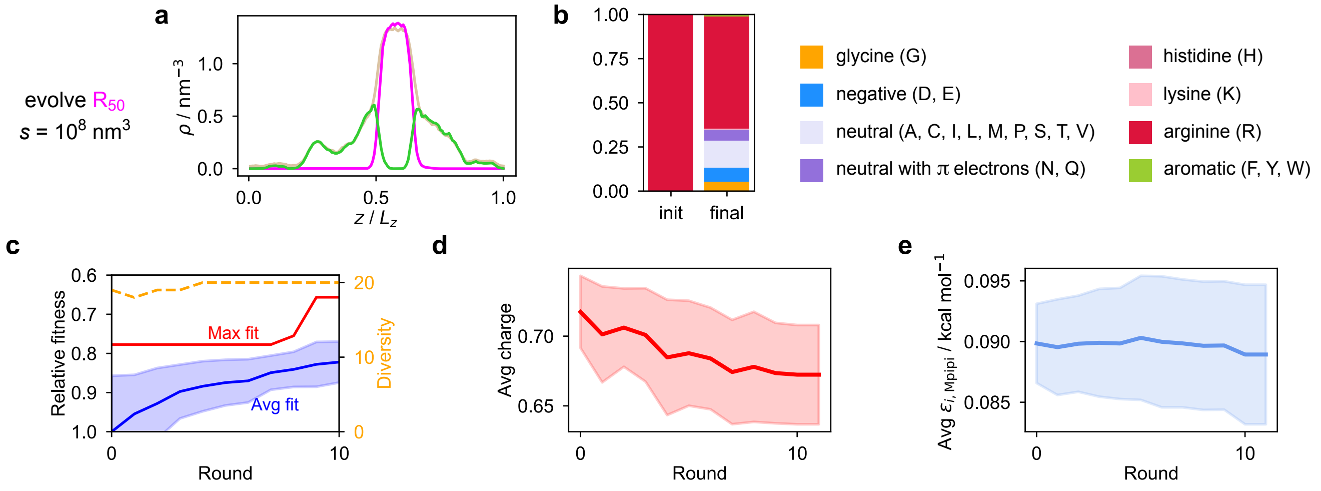

Figure S2. Genetic-algorithm run towards decreasing multiphasicity where we evolve R_{50}, using a larger value for the weighting parameter of the penalty term disfavouring full mixing ($s = 10^8$ nm^3). (*a*) Density profile of the final evolved system with maximum fitness. The pink, green and light brown curves correspond to the density profiles of (evolved) R_{50}, K_{50} and U_{10} respectively. (*b*) Changes in composition of the evolved R_{50} sequence. The final composition is averaged across all 20 sequences in the population of the final round. (*c*) Genetic-algorithm progression of the run. (*d*) Change in average charge and (*e*) $\varepsilon_{i,\mathrm{Mpipi}}$ of the evolved sequence as a function of the round number.

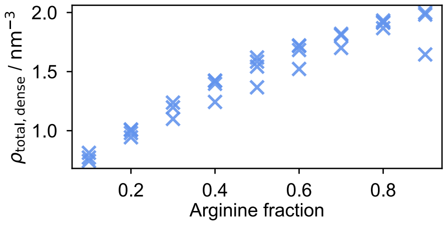

Figure S3. Density of phase-separated condensates of the protein $R_m K_{50-m}$ mixed with a charge-matched amount of U_{10} as a function of the arginine fraction of the protein. The density of the dense phase increases with arginine fraction.

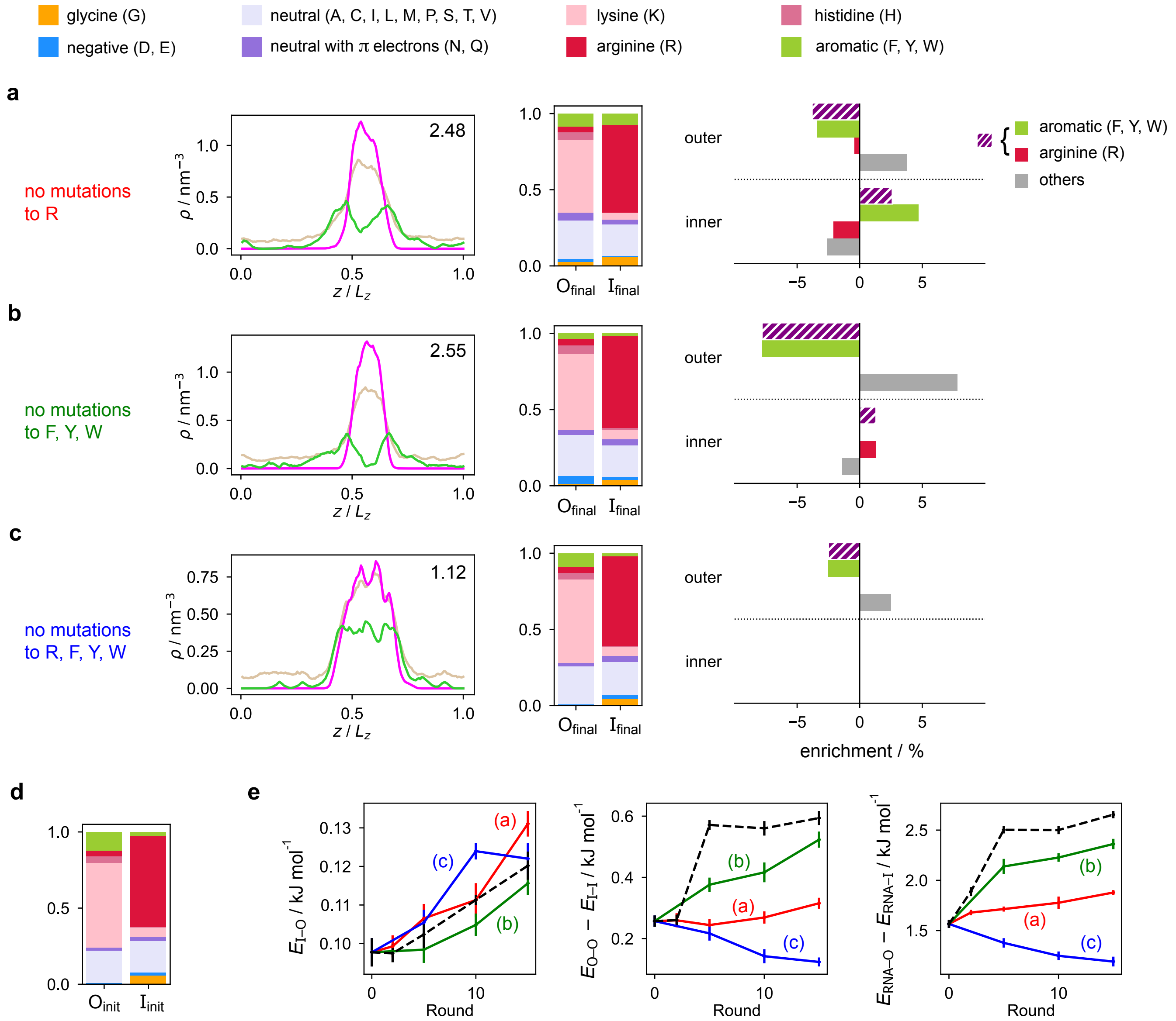

Figure S4. Density profiles of the final evolved system with maximum fitness and final composition of the evolved sequences in the genetic-algorithm runs towards increasing multiphasicity, where we evolve both sequences simultaneously while disallowing mutations to (*a*) arginine (R), (*b*) aromatic residues (F, Y, W), and (*c*) both arginine and aromatic residues. In the top right-hand corner of each density plot, we give the fitness value of the final system relative to the initial starting system [Fig. 4(*a*)]. 'O'/outer and 'I'/inner refer to the proteins concentrated in the outer and inner phases of the multilayered condensates formed. The initial compositions of both proteins are shown in (*d*) for reference. (*e*) Interaction energies between the different species within the condensate as a function of the round number in the genetic-algorithm run. Red, green and blue curves correspond to the cases in (*a*), (*b*) and (*c*) respectively. The black dashed curve corresponds to the unconstrained case presented in the main text where mutations to all residues are allowed [Fig. 4(*a*–*c*)]. The blue curve corresponding to case (*c*) does not correspond to a significant increase in fitness and the final system is not more multiphasic, so we do not necessarily expect the interaction energies to follow the same trends as the other cases. Error bars correspond to the standard deviation in the energy computed over 4 independent 100 ns-simulations.

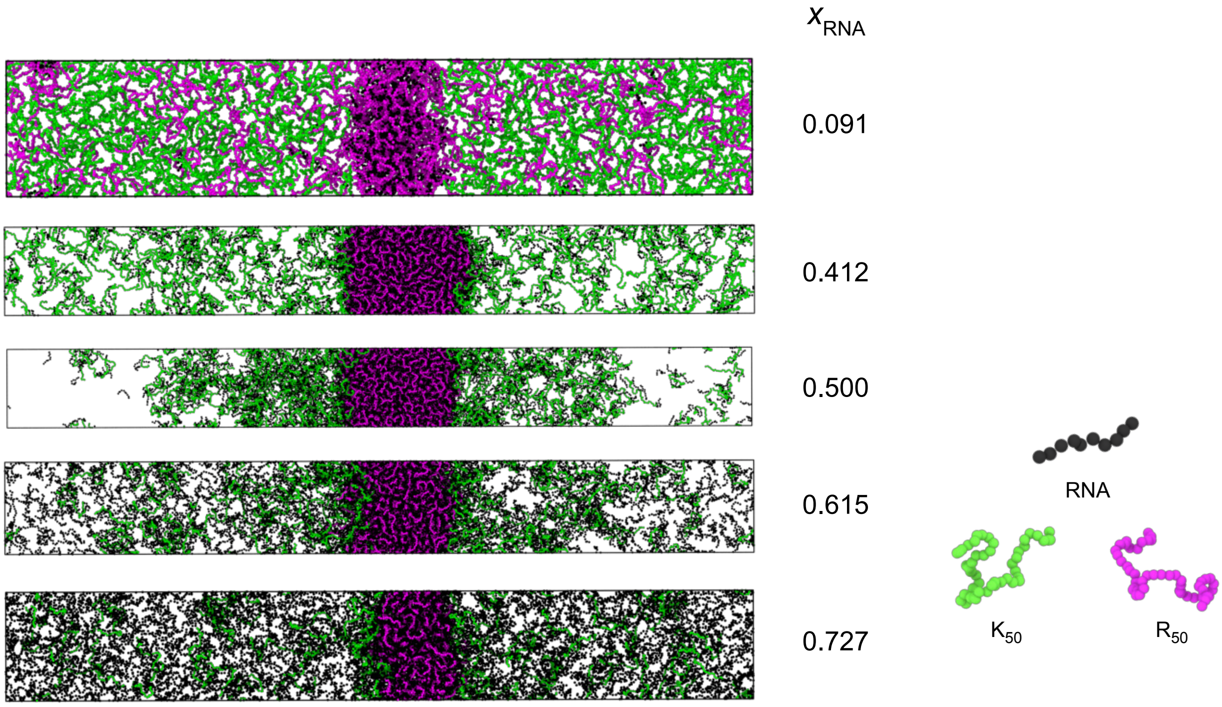

Figure S5. Variation in phase behaviour as a function of the fraction of RNA in mixtures of poly-arginine (R_{50}), poly-lysine (K_{50}) and RNA (U_{10}). In all of these mixtures, the ratio R_{50} to K_{50} is kept constant at 1:1 as the fraction of RNA of the total system is changed. Even a small amount of RNA (x_{RNA} = 0.091) can stabilise a single condensate of R_{50}, but two condensed phases occur only at $x_{RNA} \approx 0.5$ where the mixture is overall charge neutral. For larger mole fractions, the system appears to begin to favour a vapour in contact with one condensed phase with slight wetting at the interface.

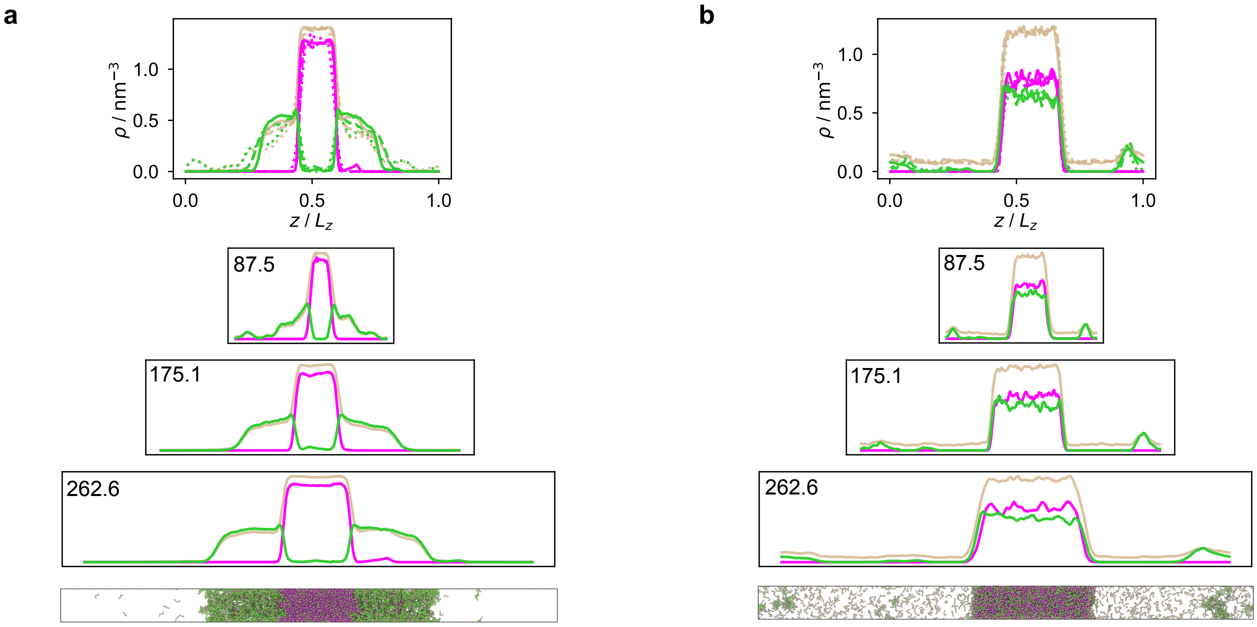

Figure S6. Finite-size scaling analysis for (*a*) the initial multilayered R_{50}, K_{50} and U_{10} system and (*b*) the final evolved system with low multiphasicity in Fig. 2(*a*). In each panel, the top figure shows the density profiles, scaled along the horizontal axis for ease of comparison, across the three different system sizes. The original system size is shown in dotted lines, and systems at two and three times the original size are shown in dashed and solid lines, respectively. We also show the unscaled density profiles for the three system sizes and a simulation snapshot of the largest system size considered. In these simulations, we keep the area of the interface constant at 10.9 nm × 10.9 nm, with the long axis increasing from 87.5 nm to 175.1 nm and 262.6 nm from top to bottom, as labelled on the upper-left corner of each density profile.

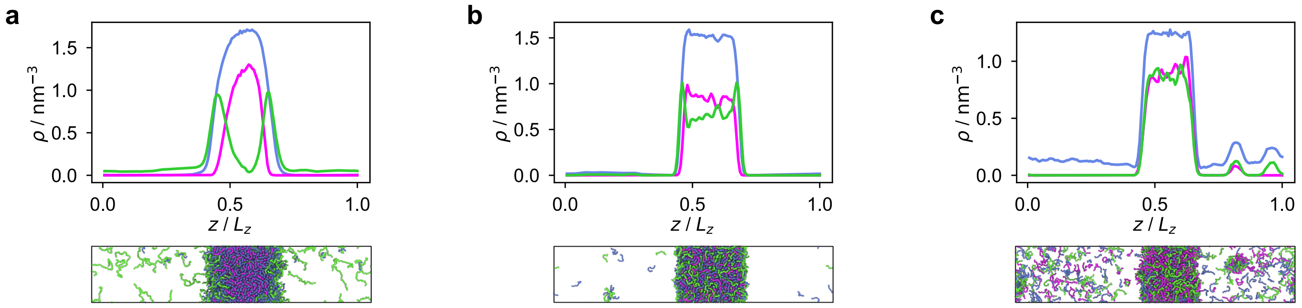

Figure S7. Density profiles of (a) the initial R_{50} (pink) and K_{50} (green) system, as well as the final evolved systems in (b) Fig. 2(a) and (c) Fig. 2(c), but with poly-guanine (G_{10}, blue) instead of poly-uracil (U_{10}). Substituting uracil for guanine, which is a purine and has stronger attractive interactions with the amino acids than uracil, maintains the relative multiphasicity of the systems, and hence we expect the trends we have reported to be similar irrespective of the choice of RNA sequence, as long as there are no base-pairing effects to consider.

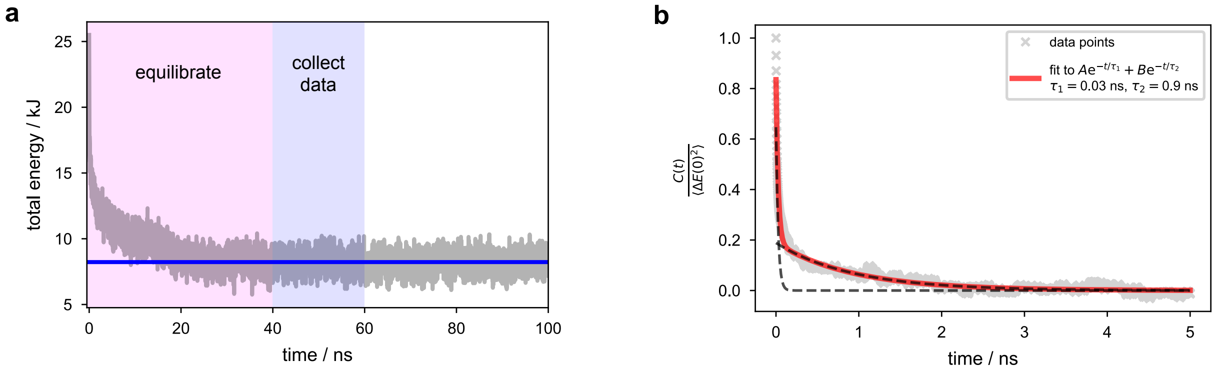

Figure S8. Convergence analysis. (a) Typical time evolution of the energy for a system immediately following a genetic-algorithm crossover and mutation, i.e. a system that is not initially at equilibrium, but evolves towards it. The blue line gives the mean of the energy computed from only the data points in the 20 ns of data collection, as indicated. This is shown for the system in Fig. 2(a). (b) Energy auto-correlation function for the multilayered R_{50}, K_{50} and U_{10} system at local equilibrium. We define the auto-correlation function as $C(t) = \langle \Delta E(t) \Delta E(0) \rangle$, where $\Delta E(t) = E(t) - \langle E \rangle$ and $\langle E \rangle$ is the mean energy averaged over all times. We have fitted the data points to the function $A \exp(-t/\tau_1) + B \exp(-t/\tau_2)$ (red curve; the individual contributions from the two exponentials are shown by the black dashed curves) to obtain characteristic decorrelation times of $\tau_1 = 0.03$ ns and $\tau_2 = 0.9$ ns. This analysis suggests that the 20 ns simulation time for density computation corresponds to a large number of decorrelated configurations.
